# A randomized clinical trial of the effectiveness of mechanical traction for sub-groups of patients with low back pain: study methods and rationale

**DOI:** 10.1186/1471-2474-11-81

**Published:** 2010-04-30

**Authors:** Julie M Fritz, Anne Thackeray, John D Childs, Gerard P Brennan

**Affiliations:** 1 Rehabilitation Agency, Intermountain Healthcare, Salt Lake City, Utah, USA; 2 Department of Physical Therapy, The University of Utah, Salt Lake City, Utah, USA; 3 U.S. Army-Baylor University, Fort Sam Houston, Texas, USA

## Abstract

**Background:**

Patients with signs of nerve root irritation represent a sub-group of those with low back pain who are at increased risk of persistent symptoms and progression to costly and invasive management strategies including surgery. A period of non-surgical management is recommended for most patients, but there is little evidence to guide non-surgical decision-making. We conducted a preliminary study examining the effectiveness of a treatment protocol of mechanical traction with extension-oriented activities for patients with low back pain and signs of nerve root irritation. The results suggested this approach may be effective, particularly in a more specific sub-group of patients. The aim of this study will be to examine the effectiveness of treatment that includes traction for patients with low back pain and signs of nerve root irritation, and within the pre-defined sub-group.

**Methods/Design:**

The study will recruit 120 patients with low back pain and signs of nerve root irritation. Patients will be randomized to receive an extension-oriented treatment approach, with or without the addition of mechanical traction. Randomization will be stratified based on the presence of the pre-defined sub-grouping criteria. All patients will receive 12 physical therapy treatment sessions over 6 weeks. Follow-up assessments will occur after 6 weeks, 6 months, and 1 year. The primary outcome will be disability measured with a modified Oswestry questionnaire. Secondary outcomes will include self-reports of low back and leg pain intensity, quality of life, global rating of improvement, additional healthcare utilization, and work absence. Statistical analysis will be based on intention to treat principles and will use linear mixed model analysis to compare treatment groups, and examine the interaction between treatment and sub-grouping status.

**Discussion:**

This trial will provide a methodologically rigorous evaluation of the effectiveness of using traction for patients with low back pain and signs of nerve root irritation, and will examine the validity of a pre-defined sub-grouping hypothesis. The results will provide evidence to inform non-surgical decision-making for these patients.

**Trial Registration:**

This trial has been registered with http://ClinicalTrials.gov: NCT00942227

## Background

### The Problem of Low Back Pain with Nerve Root Involvement

Low back pain is a common condition that is estimated to affect approximately 40% of the adult population within a 1-month time frame [1]. A majority of cases of LBP are considered "non-specific", with no clearly evident pathoanatomic cause [2]. Among the most common specific causes of LBP are disorders of the lumbar intervertebral disks resulting in irritation of the lumbar nerve roots. It is estimated that nerve root involvement accompanies approximately 10% of episodes of LBP [3], with lifetime prevalence estimates ranging from 12% - 43% [4,5]. Although LBP accompanied by nerve root involvement accounts for a relatively small percentage of all cases of LBP, the presence of nerve root involvement has been associated with an increased severity of symptoms, heightened risk of chronicity and work absence, and higher health care costs [3,6-8].

Although surgery may offer benefits for some patients with LBP and nerve root involvement [9], non-surgical management is recommended as an initial strategy in most cases [10]. However, there appears to be a wide range of non-surgical management strategies undertaken for these patients, with little standardization or evidence to inform decision-making [11]. Many patients with LBP and nerve root involvement receive physical therapy [12], yet there is currently little research examining the potential benefits of physical therapy relative to other non-surgical options. A recent study suggests there may be some benefit to receiving physical therapy compared to management by a general practitioner alone [13], however the benefits of physical therapy were not consistent across all outcomes in the study, and the results did not support the overall cost-effectiveness of physical therapy [14]. Physical therapists use a wide variety of treatments for patients with LBP and nerve root involvement [15], and sub-optimal outcomes may be at least partly attributable to the relatively sparse evidence on the specific treatment options that are most effective for these patients [16,17]. It is also unclear if there is meaningful heterogeneity in the response to different physical therapy treatment options, which would indicate a potential benefit in identifying more homogeneous sub-groups within the larger group of patients with LBP and nerve root involvement.

### Mechanical Traction as a Treatment for Low Back Pain with Nerve Root Involvement

Axial traction has been used for centuries as a treatment for LBP and was popularized by Cyriax as an intervention for patients with nerve root involvement due to lumbar intervertebral disk herniation [18]. Evidence-based guidelines and systematic reviews have generally not supported the use of traction for patients with LBP [19-21]. Despite the lack of research support, surveys indicate continued use of traction, in various forms and delivered by various providers, for patients with LBP [22-25]. The rationale offered by those who advocate the use of traction despite the lack of supporting evidence is the low methodological quality and questionable external validity of the majority of the research examining traction [26]. External validity concerns have centered on divergences between the delivery of traction in research and clinical practice, including questions about dosage parameters (traction force and duration), the use of concomitant interventions, and patient selection [27]. Specifically, traction has generally been researched as a stand-alone treatment, whereas clinicians often deliver traction in conjunction with other treatments, most often exercise interventions [28]. Clinicians also report using traction for selected sub-groups of patients with LBP, whereas research has often included more heterogeneous patient samples. The most common sub-group of patients cited by clinicians as appropriate for traction are those with LBP accompanied by signs of nerve root irritation [27,28], however the accuracy of this clinical perception has not been adequately researched.

### Preliminary Research to Identify a Sub-Group of Patients Likely to Benefit from Traction

As an initial step towards investigating if a more clinically-relevant traction protocol would result in larger treatment effects, and to explore if a specific sub-group of patients with LBP exists for whom evidence would support the use of traction, we conducted a randomized clinical trial [29]. The inclusion criteria for this trial were designed to provide a more homogeneous sample that reflected clinicians' perceptions of the appropriate sub-group of patients most likely to benefit from traction. The trial required patients to have symptoms distal to the buttock accompanied by clinical signs of nerve root irritation. Because we hypothesized that clinically important heterogeneity may exist even within this more narrowly-defined sample of patients, we secondarily examined additional baseline variables, nominated based on expert opinion and observations, for their potential to further define a traction sub-group. The trial randomized patients to one of two treatment groups, both of which received 6 weeks of treatment involving an extension-oriented treatment approach (EOTA) comprised of repeated end-range extension exercises and supplemented with manual therapy and education designed to facilitate centralization of the patient's symptoms. One group also received mechanical traction during the first two weeks of treatment, in addition to this extension-oriented approach (EOTA+traction). The traction protocol was designed to reflect common clinical practice [28] and was informed by expert clinical input [30]. The protocol used a high dosage of traction, with a sustained traction force ranging between 40%-60% of a patient's body weight, delivered for a maximum of 12 minutes during a treatment session. The delivery of traction was combined with concomitant use of the EOTA interventions designed to enhance centralization of symptoms. We provided this high-dose traction during the initial 2 weeks of traction to reflect the clinical perception that traction is most effective when delivered early in the course of treatment, followed by transition to exercise or other patient-directed activities [30].

**Figure 1:**
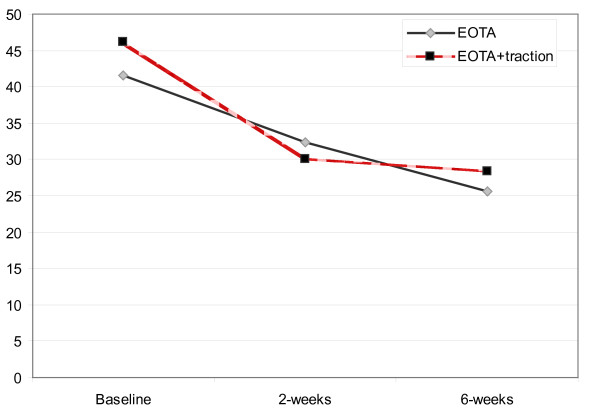
**Comparison of patients receiving an Extension-Oriented Treatment Approach (EOTA) with or without traction in the preliminary randomized trial **[29]. A significant treatment effect favoring traction was evident after 2 weeks, but not after 6 weeks.

**Figure 2:**
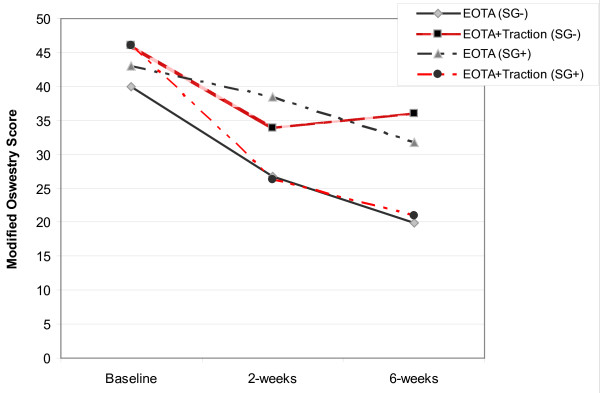
**Comparison of patients receiving an Extension-Oriented Treatment Approach (EOTA) with or without traction based on status on the sub-grouping (SG) criteria for traction in the preliminary randomized trial **[29]. A significant 3-way interaction between treatment, sub-grouping status, and time was evident after 2 and 6 weeks.

Analysis of the overall randomized trial results found a significant time x treatment interaction after 2 weeks, but not at the 6 week follow-up for the outcome of disability (Modified Oswestry questionnaire) (figure 1). The group receiving EOTA+traction experienced greater change after 2 weeks on the Oswestry questionnaire, however the magnitude of the treatment effect was at the margins of clinical significance [31] (mean difference = 7.2 points (95% CI: 0.13, 14.3)), and was no longer significant after 6 weeks. Our secondary analysis identified two baseline variables that significantly interacted with the treatment provided: the presence of peripheralization of symptoms with extension movement, and a positive crossed straight leg raise test. Analysis of the results using the presence of either of these two factors to preliminarily define a sub-group of likely traction responders (table 1) demonstrated a significant time x treatment x sub-group status, 3-way interaction at both the 2- and 6-week follow-up (figure 2). The nature of this interaction indicated that patients who met the preliminary sub-grouping criteria and received traction experienced greater change in disability across time than those who did not meet the sub-grouping criteria and received traction, or those who met the criteria but did not receive traction (figure 2). The presence of this interaction suggests the sub-grouping criteria identified in this preliminary study may represent an effect modifier specific to the use of lumbar traction (i.e., the response to lumbar traction is expected to differ based on sub-grouping status) [32]. Additional research in different patient samples is needed to validate or refute this preliminary finding [33].

**Table 1:** Definition of preliminary criteria to define a sub-group of patients likely to benefit from traction.

Criterion	Definition
**1**. Peripheralization with extension movement	Presence of peripheralization with at least one of the following extension movements during the baseline examination: single extension standing, repeated extension standing, sustained extension prone (prone on elbows), or repeated extension prone (prone press-ups). Peripheralization is judged to have occurred if a pain or paresthesia moves distally away from the spine toward the periphery, or paresthesia or a neurological sign is worsened or produced during or after the movement [48].

**2**. Positive crossed straight leg raise test	Reproduction of familiar symptoms in the symptomatic lower extremity with passive straight leg raising of the unaffected leg at an angle of 70° or less [49].

**Positive Sub-Grouping Status (SG+)**: *Either *criterion 1 or 2 is present (or both are present) **Negative Sub-Grouping Status (SG-)**: *Both *criterion 1 and 2 are absent	

## Methods/Design

### Overview of research design

**Figure 3:**
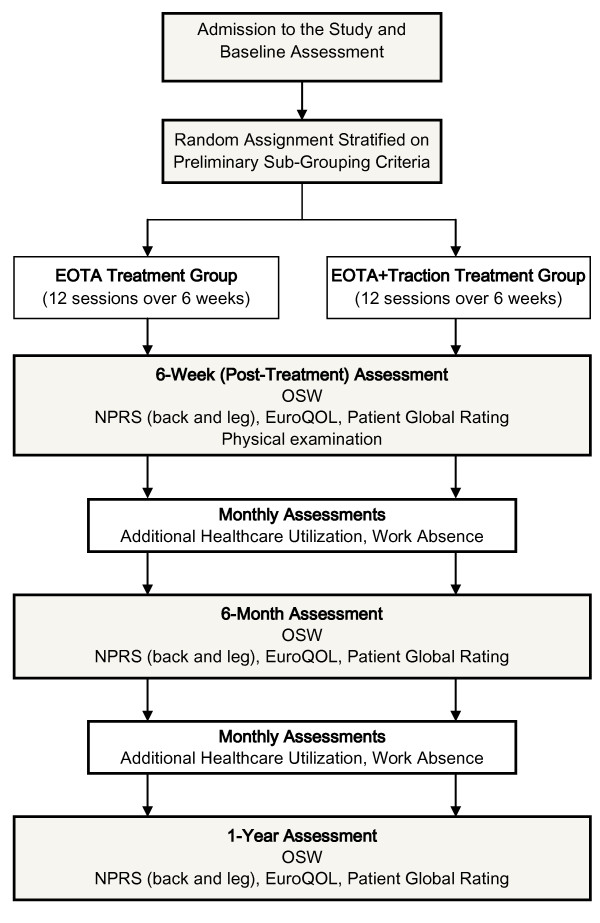
**Participant flow through the study**.

The study will be a randomized controlled trial comparing an extension-oriented treatment approach (EOTA) with or without the addition of mechanical traction. The study will include patients with signs and symptoms consistent with lumbar nerve root irritation. Randomization will be stratified based on the preliminary sub-grouping criteria (table 1). The study design and subject flow are outlined in figure 3. Subjects in each treatment group will receive 12 individual treatment sessions with a physical therapist delivered over a 6-week period. Patients randomized to the traction group will receive traction combined with the EOTA throughout the 6-week treatment period. Outcomes will be assessed at the conclusion of treatment (6 weeks), and after 6 months and 1 year. The study has been approved by the Institutional Review Boards at the University of Utah, Intermountain Healthcare, Inc., and Wilford Hall Medical Center.

### Hypotheses

1. The treatment group receiving traction in addition to an EOTA intervention will have better outcomes than the treatment group receiving the EOTA intervention without traction after 6 weeks.

2. The treatment group receiving traction in addition to an EOTA intervention will have better outcomes than the treatment group receiving the EOTA intervention without traction after 6 months and 1 year.

3. Patients who are positive on the preliminary traction sub-grouping criteria and receive traction in addition to an EOTA intervention will have better outcomes at all time points than:

a. patients who are negative on the preliminary traction sub-grouping criteria and receive traction in addition to an EOTA intervention

b. patients who are positive on the preliminary traction sub-grouping criteria and do not receive traction in addition to an EOTA intervention.

### Subject Recruitment

A total of 120 subjects will be recruited for participation. Subjects will be recruited from physician (general practice, physiatry, orthopedic surgery) and physical therapist (outpatient orthopedic) practices in Salt Lake City, Utah and San Antonio, Texas in the United States. Interested individuals from these practices will be screened for eligibility based on the inclusion/exclusion criteria. Subjects must meet each of the study's inclusion criteria, and not have any of the exclusion criteria in order to be eligible for enrollment in this study (table 2). These criteria are designed to recruit a sample of subjects with signs and symptoms of nerve root irritation without contraindications to the study treatments. Subjects over age 60, and those reporting an absence of symptoms when seated, are excluded to reduce to likelihood of including subjects whose LBP is attributable to lumbar spinal stenosis, for whom extension exercises may be contra-indicated. All potential subjects screened for eligibility will be recorded as well as the reason for ineligibility of potential subjects who are not enrolled in the study.

**Table 2:** Inclusion and exclusion criteria for participation in the study.

Inclusion Criteria	Exclusion Criteria
**1**. Chief complaint of pain and/or paresthesia in the lumbar spine with a distribution of symptoms that has extended distal to the gluteal fold into at least one lower extremity within the past 24 hours.	**1**. Known serious spinal pathology (e.g., spinal tumor, fracture, infectious disorder, osteoporosis or other bone demineralizing condition), or suspicion of serious pathology based on red flags noted in the general medical screening.
**2**. Modified Oswestry score ≥20%	**2**. Evidence of central nervous system involvement, including symptoms of cauda equina syndrome (i.e. loss of bowel/bladder control or saddle region paresthesia) or the presence of pathological reflexes (i.e. positive Babinski) in the physical examination.
**3**. Age at least 18 and less than 60 years	**3**. Patient report of a the complete absence of LBP and leg symptoms when seated
**4**. *At least one *of the following signs of nerve root compression:	**5**. Recent (within the past 2 weeks) epidural steroid injection for LBP and/or leg pain
I. Positive ipsilateral or contralateral straight leg raise test (reproduction of leg symptoms with leg raise <70°)	**6**. Current pregnancy
II. Sensory deficit to pinprick on the ipsilateral lower extremity	**7**. Known inability to comply with the treatment schedule (e.g., planned vacation, etc.)
III. Diminished myotomal strength of the ipsilateral lower extremity	
IV. Diminished lower extremity muscle stretch reflex (Quadriceps or Achilles) of the symptomatic lower extremity	

### Outcome Measures

The baseline evaluation of the subject will be conducted following confirmation of eligibility and the provision of informed consent. Demographic characteristics will be collected including the subject's age, gender, employment status, LBP history, prior treatments for LBP, and treatment expectations. Subjects' fear-avoidance beliefs regarding physical activity and work will be assessed using the Fear-Avoidance Belief Questionnaire [34], and pain catastrophizing will be evaluated with the Pain Catastrophizing Scale [35]. Measures of treatment outcome will be collected at the baseline evaluation and repeated after 6 weeks, 6 months, and 1 year.

The primary outcome measure for determining treatment effectiveness will be LBP-related disability measured using a Modified version of the Oswestry disability index (OSW) [31]. The OSW consists of 10 items assessing different aspects of pain and function related to LBP. Each item is scored from 0-5 with higher scores representing greater disability. The OSW is a recommended and widely-used measure of outcome in clinical trials evaluating treatments for patients with LBP [36], with demonstrated responsiveness to change in individuals with LBP, including those with LBP accompanied by leg pain [37,38]. Secondary outcome measures will include average low back and leg pain intensity over the past 24 hours measured using numeric rating scales [39]. Subject's will be asked to rate their current level of pain as well as the best and worst levels of pain over the past 24 hours on 11-point scales ranging from 0 "no pain" to 10 "worst imaginable pain". The mean of the three ratings will represent the average pain intensity. Separate ratings will be collected for LBP and leg pain. Additional secondary outcome measures will be the subject's global rating of change from initiation of treatment to present using a 15-level scale [40], and health-related quality of life assessed using the Euroqol (EQ-5D) [41].

Subjects' utilization of healthcare resources and associated direct healthcare costs, as well as work absence due to LBP will be recorded during the year following enrollment in the trial. The subject will be contacted by phone or e-mail on a monthly basis following enrollment. At each contact the subject will asked to report any visits to healthcare providers for the management of LBP, any diagnostic (e.g., MRI, radiographs, etc.) or interventional procedures (e.g., injection, surgery, etc.), and any prescription medication purchased for LBP. The subject will also be asked to report the number of days absent from work related to LBP. Direct healthcare costs will be computed by assigning a standardized cost to each healthcare service received based on average procedure cost data from Intermountain Healthcare's health plan.

### Randomization

Assignment of subjects to treatment groups will be performed by a research assistant following completion of all baseline evaluation procedures. Randomization will be stratified based on the presence (or absence) of the preliminary traction sub-grouping criteria. The preliminary sub-grouping criteria will be judged positive (SG+) if *either* of the two sub-grouping variables is identified during the baseline physical examination (table 1). The preliminary sub-grouping criteria will be judged to be absent (SG-) if *both* of the sub-grouping variables are absent during the baseline physical examination. Our preliminary study found 32 out of 64 subjects to be SG+ (50%), and we anticipate a similar proportion in this study. After completion of all baseline evaluation activities, the research assistant will select and open the next sealed opaque envelope appropriate to the subject's sub-grouping status. Allocation sequences will be generated in permuted block sizes of 6, 8, or 10 by study personnel prior to beginning the trial using a web-based randomization generator http://www.randomization.com. Separate sequences will be generated for each recruitment site.

### Interventions

Subjects will be randomized to one of two treatment groups. One group will receive only the EOTA intervention, the other group will receive the EOTA intervention with the addition of mechanical lumbar traction. Subjects in both treatment groups will receive a total of 12 individual physical therapy treatment sessions delivered over a 6-week treatment period. Three sessions will occur during weeks 1 and 2 of the treatment period, two sessions in weeks 3 and 4, and one session during weeks 5 and 6.

#### Extension-Oriented Treatment Approach (EOTA) Group

The EOTA intervention involves three components. The goals of each component are to promote centralization of symptoms and extension range of motion of the lumbar spine. The first component will be a series of active extension-oriented exercises (table 3). These exercises will consist of sustained and repeated extension movements performed with the subject prone or standing. The exercises may be performed with a lateral component (i.e., shifting of the pelvis in the frontal plane) if this facilitates centralization. Exercises will be progressed at the treating physical therapist's discretion, guided by the principles of maximizing centralization of symptoms and increasing extension range of motion. Not all exercises will be used for every subject, only those which promote centralization or extension range of motion. Exercise progression will be accomplished by increasing the exercise time (up to 5 minutes for sustained exercises) or repetitions (up to 30 repetitions per session), increasing the range of motion towards greater lumbar extension, and adding over-pressure once the end-range of extension range of motion is obtained. Subjects will be instructed to perform all assigned exercise activities at home, every 4-5 hours throughout the day, on days when they do not have a treatment session. Subjects will be provided a copy of an exercise instruction booklet with detailed written descriptions and pictures of the proper performance, frequency, and progression of each exercise. Subject compliance with their home exercise program will be recorded by the treating physical therapist at each treatment session.

**Table 3:** Exercise progression within the extension-oriented treatment approach.

Extension-Oriented Exercises	Goal	Options for Progression
Prone Lying	5 minutes	May use pillow to allow lumbar flexion initially if needed May shift pelvis laterally if needed Progress to full lumbar extension without pillows or lateral shift of pelvis Progress time up to 5 minutes

Prone Lying on Elbows	5 minutes	May use partial range of extension motion initially if needed May shift pelvis laterally if needed Progress to full lumbar extension with-out lateral shift of pelvis Progress time up to 5 minutes

Prone Press-Ups	30 repetitions	May use partial range of extension motion initially if needed May shift pelvis laterally if needed Progress to full lumbar extension with-out lateral shift of pelvis Progress to exhalation during the last repetition to promote increased extension Progress to therapist over-pressure during repetitions to promote increased extension Progress up to 30 repetitions

Extension in Standing	30 repetitions	May use partial range of extension motion initially if needed May shift pelvis laterally if needed Progress to full lumbar extension with-out lateral shift of pelvis Progress up to 30 repetitions

The second component of the EOTA will be subject education. Subjects will be educated to maintain the natural lordosis of the lumbar spine while sitting, and will be instructed to avoid prolonged sitting for greater than 20-30 minutes whenever possible. Additionally, subjects will be educated on the principles of symptom centralization/peripheralization, and will be given general instructions to discontinue any activities and avoid positions that cause symptoms to peripheralize, and to encourage activities or spinal positions that centralize or improve symptoms.

The third component of the EOTA will be mobilization of the lumbar spine to promote lumbar extension. The mobilization component will consist of a series of up to 20 graded oscillatory mobilizations performed with the subject prone. The physical therapist will apply a posterior-to-anterior mobilization force directed over the spinous process, or laterally over the transverse process, using a grade I - IV mobilization force as described by Maitland [42]. The therapist will select the specific grade(s) and spinal level(s) for mobilization during each treatment session based on the consideration of promoting centralization and lumbar extension range of motion.

#### EOTA + Traction Group

Subjects in the EOTA + traction group will include the EOTA components described above. Mechanical lumbar traction will be applied using a 3D ActiveTrac traction table (Chattanooga Group, Hixson, TN), a motorized, split-table, traction device with a surface that can be adjusted in 3 dimensions (flexion/extension, rotation, and side bending). Subjects will be positioned prone with the table in a neutral position in all planes. If this position causes peripheralization or an intolerable increase in symptoms, the table will be adjusted using flexion or side-bending to promote centralization of symptoms and subject comfort as needed.

Static traction will be applied for a maximum of 12 minutes total, which consists of 10 minutes at the desired intensity, plus 1-minute ramp-up and ramp-down times. The intensity of the pull will be 40-60% of the subject's body weight, adjusted based on the subject's tolerance and symptom response. After 3 minutes of traction, if the table was initially positioned in flexion or side bending, the table will be repositioned as tolerated to achieve a neutral lumbar posture. "Tolerance" will be defined as the ability to achieve a neutral posture (or as close as possible) without peripheralization of symptoms or significant increase in LBP. If the table was initially positioned using both flexion and side-bending, the table will be repositioned first to correct the side-bending accommodation, and then to correct the flexion accommodation. It may not be possible to achieve the goal of a neutral posture during each session, but the goal will be to be as close to neutral posture as tolerated by the subject each session.

At the end of traction treatment, the subject will lie still for 2 minutes prone. For subjects who end their traction treatment in a flat (neutral) position, additional extension positioning will be attempted as follows: the table will be positioned in up to 10 (of extension as tolerated, and the patients will lie still in this position for an additional 2 minutes. For patients who did not end the traction treatment in a flat (neutral) position, no extension positioning will be attempted. At the conclusion of the traction treatment, the subject will be assisted to stand and walk. Flexion, side bending, and sitting positions are to be minimized as much as possible following the traction treatment. If tolerated, the subject will be instructed to perform the same home program of extension-oriented exercises as described in the EOTA group. The mobilization component of the EOTA will be added in the 3^rd ^week for patients in the EOTA plus traction group.

#### Treatment Side Effects

A questionnaire for reporting any side effects the subject perceives to be related to treatment procedures will be completed by the subject at the 6-week follow-up. The questionnaire is based on a questionnaire used previously to examine subjects with LBP receiving spinal manipulation [43]. Subjects are asked if they experienced any commonly-reported side effects following treatment including stiffness, muscle spasm, fatigue, or increased radiating symptoms. Subjects will also be able to report side effects not specifically named. For each side effect reported, the subject is asked to report the time of onset relative to the precipitating treatment session (≤24 hours or >24 hours), the duration of the side effect symptoms (≤24 hours or >24 hours), and severity of symptoms (categorized from 1 "light" to 4 "severe").

### Physical Therapist Training and Compliance

All physical therapists will be trained by the study investigators in the treatment protocols used in this study. One training session of approximately 90 minutes will be used to review all treatment procedures. Each of the participating physical therapy clinics will be equipped with the 3D Active Trac traction table prior to initiation of the study, and physical therapists will have experience with this equipment. Each participating physical therapist will also have prior experience using an EOTA intervention. Participating physical therapists will be trained to provide treatment for subjects in either group in this study. The assignment of a study subject to a participating physical therapist will be done on a pragmatic basis (location of clinic, availability of appointment, etc.) A study treatment folder will be provided to the treating physical therapist each time he or she is assigned a new study subject. The study treatment folder will include a written protocol outline specific to the subject's group assignment, and treatment forms for each physical therapy session to allow the physical therapist to record the specific activities performed during the session and the exercises assigned to the subject for home performance. In addition, each treatment session form will require the physical therapist to record the subject's reported home exercise compliance since the last treatment session. The physical therapist's compliance with the treatment protocols will be recorded and reviewed periodically. The use of any off-protocol co-interventions will be recorded. The subject's compliance with performing the assigned home exercises will be recorded from the physical therapist's treatment forms.

### Data Integrity

The integrity of the data collected throughout the trial will be monitored by regular review of the data collection forms for missing responses, errors, or out of range values.

### Data Analysis

Intention-to-treat principles will be applied to all analyses, with all subjects analyzed with the group to which they were randomized regardless of compliance with treatment. Hypotheses 1 and 2 relate to determining the overall effectiveness of adding traction to an EOTA intervention. Linear mixed models with repeated measures will be used to analyze overall treatment effectiveness. The treating physical therapist and physical therapy clinic will be modeled as random effects. Covariates, treatment group, and treatment group by time interaction will modeled as fixed effects. The treatment group by time interaction effect will be used to examine hypotheses 1 and 2 related to the effectiveness of adding traction to an EOTA intervention. Treatment effect sizes with 95% confidence intervals will be calculated for each follow-up time point. The third hypothesis, examining effectiveness of adding traction to an EOTA intervention based on subjects' status on the preliminary sub-grouping criteria, will be examined with similar procedures with the inclusion of a 3-way, time by group by sub-grouping status interaction term. Statistical significance will be based on an alpha level of 0.05 for all analyses.

### Sample Size and Power

The sample size calculation is based on examining the differences between treatment groups after 6 weeks on the primary outcome measure (OSW), while preserving adequate power for detecting the sub-grouping effect in hypothesis 3. In our preliminary study [29] we found a treatment effect following the traction intervention of 0.47, based on a mean between group difference of 6.4 points on the OSW with a sd of 13.7, and a correlation between baseline and follow-up OSW scores of *r *= 0.57. We believe our modification of the traction protocol in the present may improve the treatment effect. A sample size of 128 subjects would provide 80% to detect a slightly larger effect size (0.50). Adjustment for the baseline OSW score will improve power versus an unadjusted comparison of means [44]. Presuming a correlation between baseline and post-treatment OSW scores of 0.57, a sample size of 86 subjects would provide 80% power for the same treatment effect [44]. To preserve adequate power to examine the interaction effect related to the sub-grouping status in hypothesis 3, we inflated the sample size based on the size of the anticipated interaction effect relative to the overall treatment effect [45]. In our preliminary study, the magnitude the interaction effect was 1.07 sd between subjects receiving traction who were SG+ compared with subjects receiving EOTA only who were SG+, and was 0.71 sd between subjects receiving traction who were SG+ and those receiving traction who were SG-. If the sub-group effect size is presumed to be at least 1.0, no further sample size inflation would be required to maintain the same power as for the overall treatment effect, while a sub-group effect size of 0.71 would require an inflation of approximately 1.5 [45]. Inflating the sample size by 1.2 results in a sample size of 104 subjects. Recruiting 120 subjects (60 per group) allows for a drop-out rate of approximately 13%. Our sample size estimate would be under-powered if a large clustering effect due to subjects within clinical sites were present [46]. We are presuming that our use of standardized protocols will make any clustering effects negligible. If this presumption proves incorrect the study could be under-powered. Conversely, our sample size calculation accounts for adjustment for baseline OSW values. We will adjust for additional baseline co-variates which may increase power. A sample size of 120 subjects provides 80% power to detect a between-group difference in low back or leg pain rating of 1.0 points presuming a sd of 2.0, and a difference in the percentage of successful patients of 25% based on dichotomization of the global rating of change scale.

## Discussion

This study will examine the effectiveness of adding a standardized protocol of lumbar mechanical traction to an extension-oriented treatment approach for individuals with LBP and nerve root irritation who are receiving non-surgical care. The study will further examine if the addition of the traction protocol is particularly beneficial in a sub-group of subjects defined by the presence of clinical examination factors identified in preliminary research. The study will examine the overall effect of the treatment received, and the interaction between treatment received and the sub-group status. This is the recommended strategy for determining if treatment effects differ across sub-groups [47]. The sample size has been adjusted to preserve adequate power for the analysis of the sub-grouping interaction effect [45]. There is currently a lack of evidence to inform clinical decision-making for the non-surgical management of LBP and nerve root irritation, even though a period of non-surgical management is recommended for most patients. The overall goal of this study is to provide evidence that can improve decision-making for these patients.

## Competing interests

We acknowledge a potential competing interest in the funding of this study, which is provided by DJO incorporated, Vista, California, USA. DJO is the parent company of Chattanooga, Inc., the manufacturer of the 3D ActiveTrac traction table used in this study.

## Authors' contributions

JF and JC conceived of this study and participated in its design. AT and GB contributed to the design of this study. AT will serve as the study coordinator. All authors have read and approved the final manuscript.

## Pre-publication history

The pre-publication history for this paper can be accessed here:

http://www.biomedcentral.com/1471-2474/11/81/prepub
